# Supplementary material for: Using large language models as a scalable mental status evaluation technique
Source: NPP Digit Psychiatry Neurosci. 2025 Nov 13;3:27. doi: 10.1038/s44277-025-00042-z (PMC12624874; doi:10.1038/s44277-025-00042-z)
Supplement: Supplementary file 1 — Appendix [file 44277_2025_42_MOESM1_ESM.docx]

**APPENDIX**

**A1 Model Selection**

**Table A1.1.** Performance of Top 5 Transformer Models.

| **Model Name (Source)** | **Model Type** | **F1 Score (%)** | **Balanced Acc (%)** |
| --- | --- | --- | --- |
| TweetEmotionEval (elonzo) | RoBERTa | 77.26 | 82.37 |
| BERT base uncased | BERT | 77.04 | 82.00 |
| TwitterSentiment (cardiffnlp) | XLM-RoBERTa | 76.92 | 81.95 |
| DistillBERT base uncased | DistilBERT | 76.81 | 81.81 |
| TwitterSentiment (cardiffnlp) | RoBERTa | 76.52 | 81.60 |

Table A1.1 displays the top 5 performing transformer models using the F1 score as the sorting metric. These scores are without data augmentation, hyperparameter tuning, or any other optimizations. Balanced accuracy is seen to monotonically increase as well as the F1 score. Interestingly, if the models are sorted by balanced accuracy performance, the top four models remain the same, and the fifth best model swaps with the sixth best model (F1: 76.50% BA: 81.72%, model type: RoBERTa). Three of the top five models are a RoBERTa model or model variant. Two are baseline models while three are models fine-tuned on tweets for another related emotion or sentiment task. Nine of the forty-eight models performed no better than chance, with an F1 and BA of 0.5 and were thus removed from the following analyses as outliers.


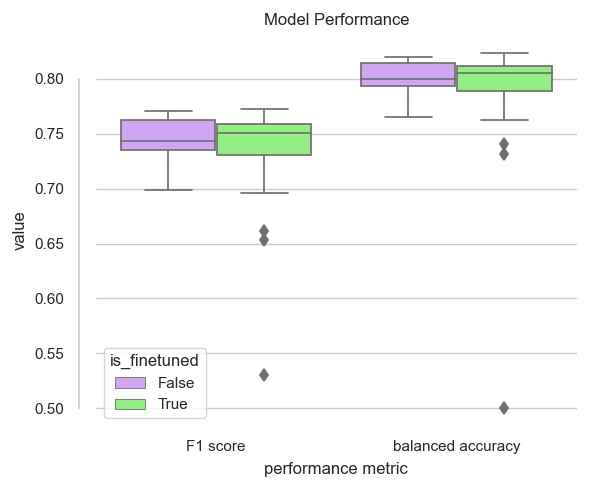


**Figure A1.** Performance of all transformer models trained during model selection. Models previously fine-tuned on related subtasks (*purple*) tended to perform slightly better on average than base models (*green*).

**Table A1.2.** Performance of classical machine learning models.

| **Languages** | **F1 Score (%)** | **Balanced Acc (%)** |
| --- | --- | --- |
| Logistic Regression | 68.12 | 75.22 |
| Multilayer Perceptron | 62.64 | 71.96 |
| Linear SVM | 62.36 | 70.64 |
| XGBoost | 61.47 | 70.96 |
| RBF SVM | 57.76 | 69.35 |
| AdaBoost | 55.28 | 67.18 |
| Decision Tree | 54.82 | 64.73 |

**A2 Back-Translation Data Augmentation Model Performance**


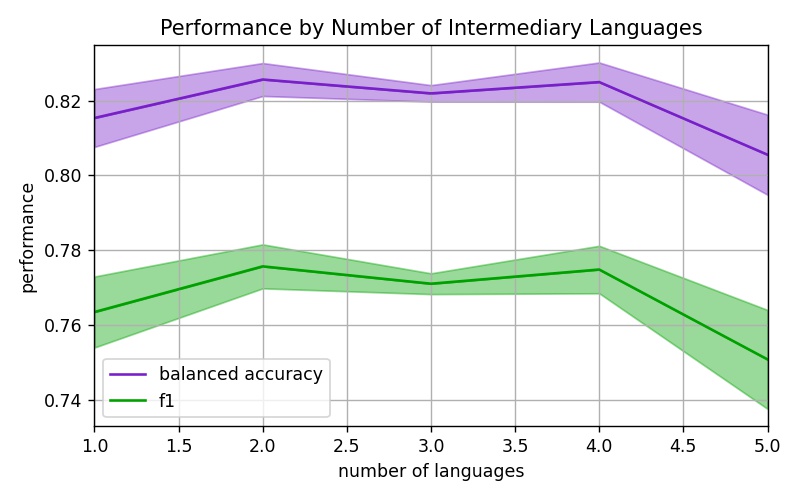
**Table A2.** Performance of the top 5 language combinations.

| **Languages** | **F1 Score (%)** | **Balanced Acc (%)** |
| --- | --- | --- |
| Turkish + Danish | 79.10 | 83.64 |
| Bengali + Turkish + Finnish + Spanish | 78.55 | 83.38 |
| Bengali + Uralic + Finnish + Portuguese | 78.24 | 83.11 |
| Bengali + Turkish + Dutch + Estonian | 78.18 | 83.09 |
| Spanish | 78.15 | 82.97 |

**Figure A2.** Effect of the number of intermediary languages on model performance in back-translation data augmentation. The solid line is the mean with the surrounding standard deviation. Models performed best with two intermediary languages and worse with five or more languages.

**A.3 Final Hyperparameters**

**Table A3.** Final hyperparameters used after hyperparameter tuning.

| **Parameter** | **Value** |
| --- | --- |
| Learning Rate | 2.528e-5 |
| Random Seed | 33 |
| Batch Size | 8 |
| Epochs | 1 |
